# Risk of major depressive disorder in spouses of cancer patients in Japan: A cohort study using health insurance‐based claims data

**DOI:** 10.1002/pon.5403

**Published:** 2020-05-26

**Authors:** Tatsuo Akechi, Izumi Mishiro, Shinji Fujimoto, Katsuhito Murase

**Affiliations:** ^1^ Department of Psychiatry And Cognitive‐Behavioral Medicine Nagoya City University Graduate School of Medical Sciences Nagoya Japan; ^2^ Japan Medical Office Takeda Pharmaceutical Company Limited Tokyo Japan

**Keywords:** administrative claims, healthcare, cancer, depressive disorder, major, epidemiology, oncology, spouses

**Key Points**

- Using a Japanese medical insurance database, we determined the risk of major depressive disorder (MDD) in spouses of newly diagnosed cancer patients compared with patients and with spouses of cancer‐free individuals.
- Spouses of cancer patients had half the cancer patients' risk of MDD (hazard ratio 0.42 [95% CI 0.36‐0.48]).
- When compared with spouses of individuals without cancer or MDD, spouses of patients with cancer (but not MDD) had an elevated risk of MDD (1.18 [1.03‐1.34]).
- Spouses' risk of MDD greatly increased (2.32 [1.50‐3.61]) if cancer patients also developed MDD.
- Greater mental health support is required for both cancer patients and their spouses.

## 1 BACKGROUND

A cancer diagnosis is a highly stressful event that can lead to psychological disorders, including depression, in the patient. Depressive symptoms occur in 6% to 40% of cancer patients, and up to 15% of cancer patients are diagnosed with major depressive disorder (MDD).^1^ However, cancer also affects family members, especially spouses, who share a close personal relationship with the patient and are often the primary caregiver.^1^ A recent meta‐analysis reported that the prevalence of depression or depressive symptoms (determined by a range of psychometric tools) in caregivers of cancer patients was 42.3%.^2^ Although many studies have documented a high level of psychological distress in spouses or caregivers of cancer patients, sometimes surpassing that of the patients themselves, few studies have examined whether spouses are actually diagnosed with MDD or meet Diagnostic and Statistical Manual of Mental Disorders criteria for MDD.^3^, ^4^, ^5^, ^6^, ^7^ Further, no previous study has determined the hazard ratio (HR) of an MDD diagnosis in spouses relative to cancer patients or relative to spouses of cancer‐free individuals.

We recently conducted a matched cohort study of Japanese workers and their families enrolled in an insured medical services database, in which the risk of MDD in cancer patients in the year after a cancer diagnosis was 2.96‐fold greater than in cancer‐free individuals.^8^ This exploratory analysis aimed to identify the risk of MDD in spouses of cancer patients compared with spouses of a cancer‐free group, and compared with cancer patients. We hypothesized that the risk of MDD in spouses of cancer patients is comparable to that of a cancer patient, but higher than the risk in spouses of cancer‐free people.

## 2 METHODS

This was an exploratory analysis of a previously described matched cohort study^8^ using data on individuals enrolled in the JMDC Inc. (Tokyo, Japan) insured medical services database between January 2011 and September 2018. Because the study used anonymized information, institutional ethics approval and informed consent were not required, in accordance with Ethical Guidelines for Medical and Health Research Involving Human Subjects in Japan.

As described,^8^ adult cancer patients who were newly diagnosed with cancer were matched 1:10 to cancer‐free individuals according to age, sex, insurance membership category, and index month. Both cancer and cancer‐free groups had no MDD diagnosis between 6 and 12 months before the index month (full inclusion criteria are listed in Table S1). This analysis included spouses of individuals in the matched cohort who did not have MDD diagnosis between 6 and 12 months before the index month. As same‐sex marriages are not legally recognized in Japan, only heterosexual couples were included. Diagnoses were based on International Statistical Classification of Diseases and Related Health Problems, 10th revision codes (cancer: C00–C95; MDD: F32–F33).

We measured the time to MDD diagnosis from 6 months before to 12 months after the index month in cancer patients with spouses, in the spouses of those cancer patients, in cancer‐free individuals with spouses, and in the spouses of those cancer‐free individuals.

Multivariate analysis was performed using a Cox proportional hazards model (SAS version 9.4; SAS Institute, Cary, North Carolina) to estimate the HR for time to MDD diagnosis in the following: spouses of cancer patients without MDD, spouses of cancer patients with MDD, and spouses of cancer‐free individuals with MDD relative to spouses of cancer‐free individuals without MDD, with covariates of sex/age of the spouse, insurance membership category of the spouse, and age of cancer/cancer‐free individual (Model 1); and spouses of cancer patients, spouses of cancer‐free individuals, and cancer‐free individuals relative to cancer patients, with covariates of sex/age of the spouse and insurance membership category of the spouse (Model 2).

## 3 RESULTS

This analysis included 16,936 married couples from the cancer group and 168,831 married couples from the cancer‐free group (Table 1). Slightly more than half of spouses were men (51.8% and 51.5% in the cancer and cancer‐free spouse groups, respectively); the same percentages (51.8%, 51.5%) of spouses were insured workers. The mean age was 53.2 years in both spouse groups and 53.6 years in both patient groups. Approximately 80% of patients and spouses were aged 40 to 64 years.

**TABLE 1:** Background and characteristics of cancer patients and their spouses, and cancer‐free individuals and their spouses

Variable	Cancer N=16,936 couples		Cancer‐free N=168,831 couples	
	Patients	Spouses	Individuals	Spouses
Sex				
Male	8171 (48.2)	8765 (51.8)	81,956 (48.5)	86,875 (51.5)
Female	8765 (51.8)	8171 (48.2)	86,875 (51.5)	81,956 (48.5)
Age				
Mean (SD)	53.6 (9.6)	53.2 (9.3)	53.6 (9.6)	53.2 (9.4)
Median (range)	54.0 (23–74)	54.0 (23–74)	54.0 (20–74)	54.0 (20–74)
<40	1358 (8.0)	1394 (8.2)	13,640 (8.1)	14,132 (8.4)
40–64	13,350 (78.8)	13,659 (80.7)	132,555 (78.5)	135,789 (80.4)
≥65	2228 (13.2)	1883 (11.1)	22,636 (13.4)	18,910 (11.2)
Worker or dependent				
Worker	8158 (48.2)	8778 (51.8)	81,814 (48.5)	87,017 (51.5)
Dependent	8778 (51.8)	8158 (48.2)	87,017 (51.5)	81,814 (48.5)

*Note*: Data are n (%), unless otherwise noted.

Spouses of patients with cancer but not MDD had a slightly, but significantly, elevated risk of MDD relative to spouses of individuals with neither cancer nor MDD (HR 1.18 [1.03‐1.34]) (Table 2). However, spouses of individuals without cancer but with MDD had approximately twice the risk of MDD compared with spouses of cancer‐free individuals without MDD (HR 2.10 [1.63‐2.73]). Spouses of patients with both cancer and MDD had the highest risk of MDD (HR 2.32 [1.50‐3.61]).

**TABLE 2:** Multivariate analyses of time to MDD within 12 months in cancer patients and their spouses, and in cancer‐free individuals and their spouses

Variable	Reference	Category	Hazard ratio (95% CI)
Model 1			
Group	Spouses of cancer‐free individuals without MDD	Spouses of cancer patients without MDD	1.18 (1.03–1.34)
		Spouses of cancer‐free individuals with MDD	2.10 (1.63–2.73)
		Spouses of cancer patients with MDD	2.32 (1.50–3.61)
Sex, age of spouse	Male, 40–64	Male, <40	0.90 (0.71–1.15)
		Male, ≥65	0.69 (0.55–0.88)
		Female, <40	1.29 (0.73–2.27)
		Female, 40–64	1.53 (0.91–2.56)
		Female, ≥65	1.61 (0.92–2.83)
Worker or dependent spouse	Worker	Dependent	0.66 (0.40–1.11)
Age of cancer patient or cancer‐free individual	40–64	<40	1.23 (1.00–1.49)
		≥65	0.96 (0.82–1.13)
Model 2			
Group	Cancer patients	Spouses of cancer patients	0.42 (0.36–0.48)
		Spouses of cancer‐free individuals	0.35 (0.32–0.38)
		Cancer‐free individuals	0.32 (0.29–0.35)
Sex, age of spouse	Male, 40–64	Male, <40	1.05 (0.90–1.22)
		Male, ≥65	0.65 (0.57–0.74)
		Female, <40	1.02 (0.68–1.53)
		Female, 40–64	0.94 (0.64–1.38)
		Female, ≥65	0.87 (0.58–1.31)
Worker or dependent spouse	Worker	Dependent	1.19 (0.81–1.75)

Abbreviations: CI, confidence interval; MDD, major depressive disorder.

Male spouses aged ≥65 years were at lower risk of MDD compared with middle‐aged (40‐64 years) male spouses (HR 0.69 [0.55‐0.88]) (Table 2). Spouses of patients aged <40 years were at elevated risk of MDD, although the HR was not statistically significant (HR 1.23 [1.00‐1.49]). Although the effect of sex was not specifically tested, female spouses tended to have higher risk of MDD than male spouses (Table 2).

Compared with cancer patients, spouses of cancer patients had less than half the risk of MDD (HR 0.42 [0.36‐0.48]). The risk of MDD in spouses of cancer patients was only slightly higher than the risk in cancer‐free individuals (HR 0.32 [0.29‐0.35]) or their spouses (HR 0.35 [0.32‐0.38]) relative to cancer patients (Table 2).

## 4 DISCUSSION

In this Japanese database analysis, spouses of cancer patients were at elevated risk of MDD diagnosis compared with spouses of cancer‐free individuals, but at approximately half the risk of cancer patients. Given that the level of psychological distress and depressive symptoms of spouses is similar to or higher than that of the cancer patients,^1^, ^2^ this finding suggests that MDD in spouses of cancer patients may be underdiagnosed. Because both spouses and physicians are highly focused on the care of the cancer patient, the well‐being of the spouse is often overlooked. In Japan, attitudinal reasons may also contribute to why spouses do not seek medical treatment for depressive symptoms.^9^

Interestingly, if an individual had MDD, with or without cancer, their spouse had more than twice the risk of also developing MDD compared with spouses of individuals without MDD or cancer. This observation is supported by previous research indicating that levels of depressive symptoms in spouses are often correlated.^10^ Alternatively, spouses of patients with MDD may be more willing to seek medical help for their own symptoms or may be proactively examined by the physician who is treating their husband or wife for MDD.

A key strength of this analysis was that patients and spouses were a subset of a large matched cohort of cancer and cancer‐free patients derived from a nationwide health insurance database, although married couples in the cancer group were not specifically matched to couples in the cancer‐free group. Spousal psychological stress is highest during the terminal stages of cancer; however, our study focused on the first year after a cancer diagnosis, when shock and adjustment to the diagnosis are more likely to contribute to distress than coping with the patient's physical deterioration and impending death. The large sample size allowed us to consider possible interactions between cancer and MDD in patients and their effect on the risk of MDD in spouses.

### 4.1 Study limitations

The effect of other factors, such as cancer type, stage, and prognosis, spousal physical health problems, caregiver status, absences from work, familiarity with mood disorders/MDD, or the quality of the couple's relationship were not evaluated. In addition, there were few female workers among the couples that were included in the analysis. Finally, the diagnosis criterion for this study was an actual clinical diagnosis of MDD; however, we could not determine from the database how the diagnosis was made (ie, clinical evaluation, structured interview) or whether it was made by a mental health professional or other physician.

### 4.2 Clinical implications

Greater mental health support, including appropriate diagnosis and treatment of MDD, is required not only for cancer patients, but also for their spouses.

## 5 CONCLUSIONS

This analysis suggests that MDD may be underdiagnosed in spouses of cancer patients in Japan, despite the presumed high level of spousal distress.

## CONFLICT OF INTEREST

T. A. has received lecture fees from Astellas, AstraZeneca, Daiichi‐Sankyo, Dainippon‐Sumitomo, Eisai, Hisamitsu, Kyowa‐hakko Kirin, Kyowa, Eli Lilly, MSD, Meiji‐seika Pharma, Mochida, Mundipharma, Otsuka, Pfizer, Shionogi, Terumo, and Tsumura, and has received research funds from Daiichi‐Sankyo, Eisai, FUJIFILM RI Pharma, Eli Lilly, MSD, Novartis, Otsuka, Shionogi, and Tanabe‐Mitsubishi. I. M., S. F., and K. M. are employees of Takeda Pharmaceutical Company Limited.

## Supporting information


**Table S1** Inclusion criteria for the cancer group and the cancer‐free group.

## Data Availability

The data that support the findings of this study are available from JMDC Inc. but were used under licence for the current study; therefore, restrictions apply and the data are not publicly available. For inquiries about access to the data set used in this study, please contact JMDC (https://www.jmdc.co.jp).
