# Supplementary material for: Risk of major depressive disorder in spouses of cancer patients in Japan: A cohort study using health insurance‐based claims data
Source: Psychooncology. 2020 May 26;29(7):1224–7. doi: 10.1002/pon.5403 (PMC7384032; doi:10.1002/pon.5403)
Supplement: Supplementary file 1 — Table S1 Inclusion criteria for the cancer group and the cancer‐free group. [file PON-29-1224-s001.DOCX]

SUPPORTING INFORMATION

**Table S1** Inclusion criteria for the cancer group and the cancer-free group

| **Group** | **Inclusion criteria** |
| --- | --- |
| Cancer group | - Aged 18–74 - New cancer diagnosis^†^ between January 2012 and September 2017 - No cancer diagnosis^†^ during the 12 months before index month - ≥2 cancer diagnoses^†^ at the same site within 3 months of index month - No MDD diagnosis^‡^ 6–12 months before index month - Continuous health insurance enrollment for ≥12 months before and ≥12 months after index month |
| Cancer-free group | - Aged 18–74 - No cancer diagnosis^†^ between January 2011 and September 2018 - No MDD diagnosis^‡^ 6–12 months before index month - Continuous health insurance enrollment for ≥12 months before and ≥12 months after index month |

^†^Cancer diagnosis based on ICD-10 codes C00–C95.

^‡^MDD diagnosis based on ICD-10 codes F32–F33.

ICD-10 = International Statistical Classification of Diseases and Related Health Problems, 10th revision; MDD = major depressive disorder.
